# Sex as an important factor in nanomedicine

**DOI:** 10.1038/s41467-021-23230-9

**Published:** 2021-05-20

**Authors:** Mohammah Javad Hajipour, Haniyeh Aghaverdi, Vahid Serpooshan, Hojatollah Vali, Sara Sheibani, Morteza Mahmoudi

**Affiliations:** 1 Department of Radiology and Precision Health Program, Michigan State University, East Lansing, MI US; 2 Wallace H. Coulter Department of Biomedical Engineering, Emory University School of Medicine, Georgia Institute of Technology, Atlanta, GA US; 3 Department of Pediatrics, Emory University School of Medicine, Atlanta, GA US; 4 Children’s Healthcare of Atlanta, Atlanta, GA US; 5 Department of Anatomy and Cell Biology and Facility for Electron Microscopy Research, McGill University, Montreal, QC Canada

**Keywords:** Drug delivery, Nanomedicine

## Abstract

Nanomedicine has demonstrated substantial potential to improve the quality and efficacy of healthcare systems. Although the promise of nanomedicine to transform conventional medicine is evident, significant numbers of therapeutic nanomedicine products have failed in clinical trials. Most studies in nanomedicine have overlooked several important factors, including the significance of sex differences at various physiological levels. This report attempts to highlight the importance of sex in nanomedicine at cellular and molecular level. A more thorough consideration of sex physiology, among other critical variations (e.g., health status of individuals), would enable researchers to design and develop safer and more-efficient sex-specific diagnostic and therapeutic nanomedicine products.

## Abstract (web summary)

Given the early failing of therapeutic nanomedicine in the clinic the importance of understanding bio-nano interactions and biosystem parameters has gained much attention. Here, the authors review the work to date that looks at the impact of sex on the applications and outcomes of nanomedicine.

## Introduction

According to the British Standards Institute, nanotechnology refers to “intentional design, characterization, production, and applications of materials, structures, devices, and systems by controlling their size and shape in the nanoscale range (1–100 nm)”^1^. Nanomedicine employs nanotechnology for medical applications including diagnosis and treatment of diseases^2^. As an example, for drug delivery, nanomedicine aims to design and develop biocompatible nanoparticles (NPs) that can protect payloads from degradation and unwanted interactions with biosystems (e.g., immune cells and biological barriers) and deliver them to the desired biosystems (e.g., cells)^3^. The purpose of this review is to explore the relevance of sex at cellular and molecular level for the laboratory and clinical research in the general field of nanomedicine.

Although the emergence and development of nanomedicine have demonstrated promising (and sometimes even tremendous) positive results in in vitro environmental and animal studies, therapeutic nanomedicines such as those for cancer faced significant translational challenges. More specifically, a 2019 clinical trial analysis of 75 cancer nanomedicines, that were registered through clinicaltrials.gov database, revealed that cancer nanomedicine products have had a success rate of 14% in phase 3 trials, which largely reflects the efficacy of nanomedicine products^4^. Remarkably, the trials of some nanomedicines have failed to show any improvement in pharmacokinetics or drug accumulation within tumor tissues, as compared to the parent drugs^4^.

These disheartening clinical translational results are driven at least in part by neglect and/or a failure to fully understand several crucial/influential factors (e.g., sex) at play in both nano- and bio-systems and in nano-bio interactions^3,5,6^. Therefore, identifying and gaining a much deeper understanding of these factors is an essential step in improving successful clinical translation of nanomedicine products.

### Challenges to the identification of critical factors in safe/effective nanomedicines

Monoclonal antibodies failed their first decade of clinical trials owing to their murine-derived origin; only when humanized/human monoclonals were used did clinical trials succeed. Based on current evidence^7,8^, nanomedicine may be following a similar trajectory, but perhaps for different reasons. It is essential, therefore, that all factors critical to therapeutic efficacy–including differences in biological cellular responses between males and females—be robustly considered and incorporated into both laboratory and clinical research. This will enable development of more effective drug delivery systems and more reliable diagnostics and therapeutics.

The importance of sex as a biological variable in laboratory and clinical experiments has been recognized by the biomedical research community since the 1990s^9^. However, most researchers are still using only one sex of biosystems (e.g., cells, tissues, or animals) and applying the results to both males and females. With the emergence of nanomedicine, the importance of sex in biomedical research has become even more obvious. In a recent review article, Liyod-Parry et al.^10^ provide a comprehensive overview of the role of nanotechnology and nanomedicine in women’s health research. The authors present both the benefits and shortcomings of the application of nanomedicine to both laboratory-based research and clinical trials. Most importantly, in recent years, the importance of sex at the cellular level has also been recognized in biomedical research. Nevertheless, it is still the case that only a small number of published articles report the sex of biosystems used in their study^11^.

**Fig. 1:**
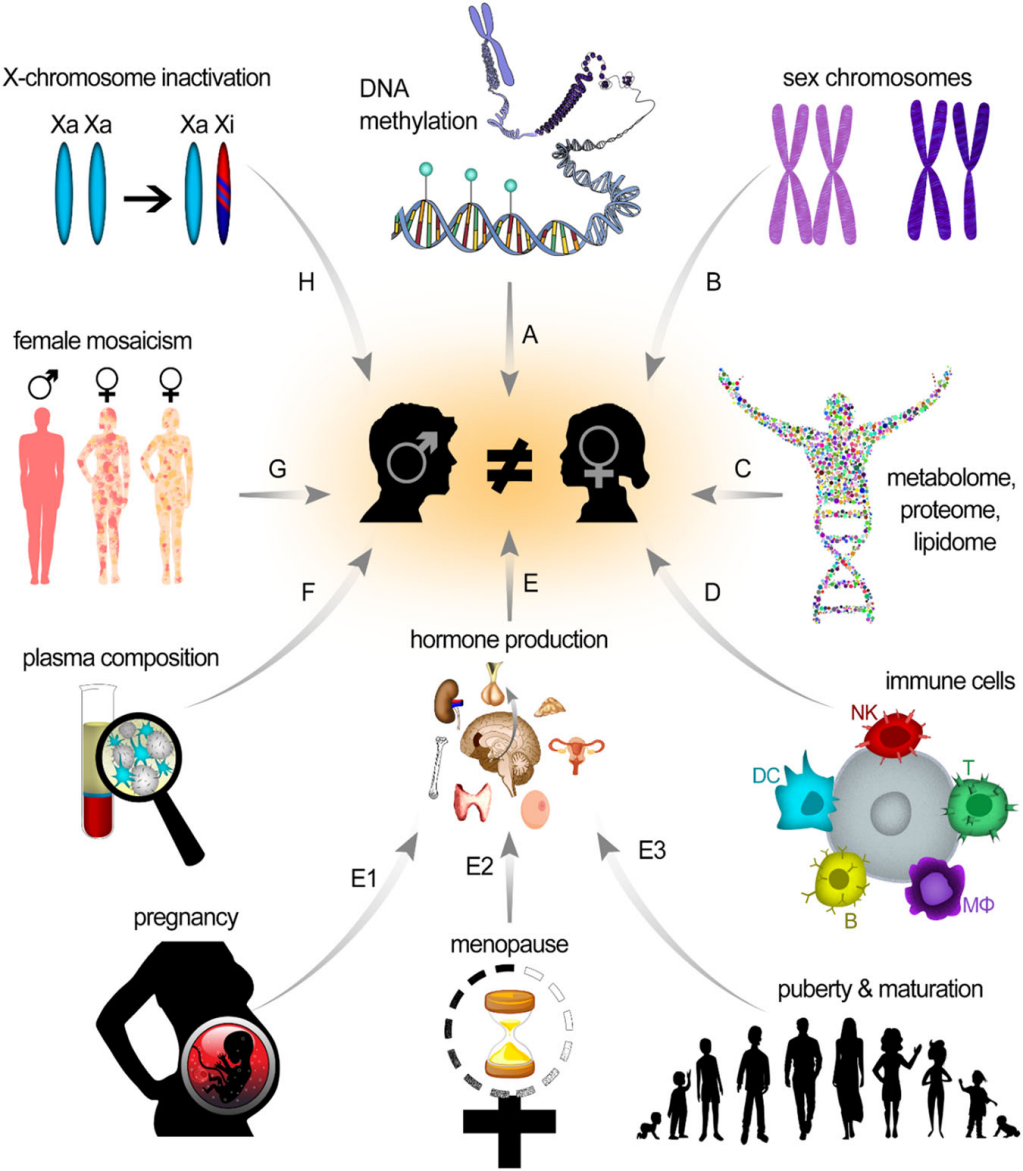
Sex-dependent physiological differences. Schematic representation of male/female differences in (**A**) DNA methylation, (**B**) sex chromosomes, (**C**) metabolome, lipidome, and proteome, (**D**) immune cells, (**E**) hormone production (during E_1_) pregnancy, E_2_ menopause, E_3_ puberty, (**F**) plasma composition, (**G**) mosaicism, and (**H**) X-chromosome inactivation.

This review focuses on how sex-dependent physiological differences (Fig. 1) can affect the interaction of NPs with biosystems. Throughout this report, the term “sex” refers to the biological trait and not gender, the social identity of individuals. The terms sex and gender might be used exchangeably, but they have different meanings. According to Canadian Institutes of Health Research (CIHR), sex refers to biological attributes of humans and animals, including physical features, chromosomes, gene expression, hormones, and anatomy, while gender refers to socio-cultural factors including socially constructed roles, behaviors, expressions, and identities.

Although only a handful of nanomedicine studies have focused on the sex-dependent effects of NPs, results indicate that the same NPs have different therapeutic and toxic impacts in male and female animals. For example, PEG-coated gold NPs show different toxic effects in male and female mice, producing more severe kidney damage in females, but higher liver toxicity in males^12^. In another study, amorphous silica NPs triggered more-severe inflammation and lung tissue damage in female rats compared to male rats^13^.

### Relevance of sex differences in cellular NP interactions and nanomedicine

With the emergence of nanomedicine, a wide range of NPs has been developed for diagnostics/therapeutics application^14,15^. However, despite the extensive deployment of therapeutic nanomedicines in both the laboratory and clinical trials, they have had very limited success in reaching clinical practice. The safe and effective use of therapeutic nanomedicine remains a growing concern among biomedical and clinical researchers^7,8,16^. Among the many reasons for the frequent failure of clinical trials involving nanomedicine is our lack of a deep understanding of the mechanisms of interaction between NPs and body fluids, extracellular matrix, and cellular components. Another important stumbling block is the fact that most laboratory and clinical studies involving cellular interactions with nanomaterials do not take the significance of sex (or, indeed, many other relevant factors^6^) into account. This is more obvious in studies involving diseased cells and tissues.

Recent studies provide convincing evidence that sex differences can alter NP efficacy at the cellular level^17^. For example, the level and pathway of NP uptake and intracellular trafficking in human amniotic mesenchymal stem cells and cancer cells are strongly sex dependent; in addition, the composition of the biomolecular/protein corona (i.e., a layer of various biomolecules that forms on the surface of NPs upon contact with a biological fluid^18^) is affected by sex-specific paracrine factors^17^. Furthermore, it has been found that toxic silver NPs significantly alter the bacterial species harbored in male zebrafish, but not in females^19^. It is clear that challenges to the progress of NP-based nanomedicines will not be overcome until biological variable aspects are taken into consideration, including alteration of the biomolecular/protein corona on the surface of NPs and different structural and molecular responses to functionalized NPs by male and female cells.

It is noteworthy that the extent of sex-related differences in biosystem responses to NPs/drugs depends strongly on the type of disease and tissue involved. Sex difference of the whole organism at the physiological level is one of the most important biological variables determining the incidence, prevalence, and severity of many diseases, including cancer, neurodegenerative, cardiovascular, autoimmune, and psychological diseases^20–26^. For example, in cancers, sex significantly influences tumor incidence, growth, cellular and molecular phenotypes, and therapeutic efficacy^27–29^. This is at least partially due to sex-related genetic differences: abnormal reactivation of X-linked genes^30^, differences in duplication/deletion of segments on X/Y chromosomes^28^, differences in mutated genes in tumors^31^, transcription factor-binding patterns^29^, and sex-specific roles of hormones^32^. Drugs also have different pharmacodynamic and pharmacokinetic patterns in males and females and, therefore, exert sex-dependent therapeutic and toxic effects^33–35^. Male and female cells of different origin may respond differently to the same NPs. For example, quantum dots have higher uptake in female than in male human amniotic stem cells; however, the same particles have shown lower uptake in female salivary gland primary cells compared to their male counterparts^17^.

### Biological identity of NPs determined by the biomolecular/protein corona

Once exposed to biological fluids, the surface of NPs becomes rapidly covered with lipids, metabolomes, proteins, and other biomolecules; this biomolecular/protein corona alters the original surface of the NPs and gives them a new biological identity^18^. Most studies in nanomedicine have not properly reported/considered biological variable factors in the in vitro and in vivo microenvironment that determine the fate, safety, and efficacy of NPs in both animal and human clinical trials^6,8^. Corrective efforts have mainly involved: (1) identifying important biological variable factors in the NP microenvironment; (2) improving NP toxicity assays; (3) optimizing in vitro protocols mimicking in vivo conditions; and (4) more-accurate computational modeling approaches to identify NP mechanisms of action^8,36,37^.

Human plasma is the most suitable biological fluid to study biomolecular/protein corona. One of the major sources of misprediction of the biological safety and efficacy of NPs is conflicting biomolecular/protein corona data, originating from the use of various biological fluids, including (1) aqueous non-biological fluids, (2) semi-biological fluids such as cell culture media, and (3) animal serum/plasma. Even when using human plasma, characteristics of the donor (e.g., sex, age, and health status) can significantly affect the composition of the biomolecular/protein corona. Unfortunately, much of the current literature on the corona does not report the details of the plasma used; in fact, in most cases, pooled plasmas from multiple individuals are employed^6^. Exposure of NPs to proteins in the plasma of healthy individuals vs. patients with cancerous conditions significantly affects the composition of biomolecular/protein coronas, pointing to NPs’ therapeutic and diagnostic properties^38–40^. It has been shown that changes in plasma composition during the disease development process substantially alter corona composition and thus NP biological function^38,41–43^. Other recent findings have revealed the role of sex-dependent secretion of paracrine factors in NPs’ interactions with cells^17^.

**Fig. 2:**
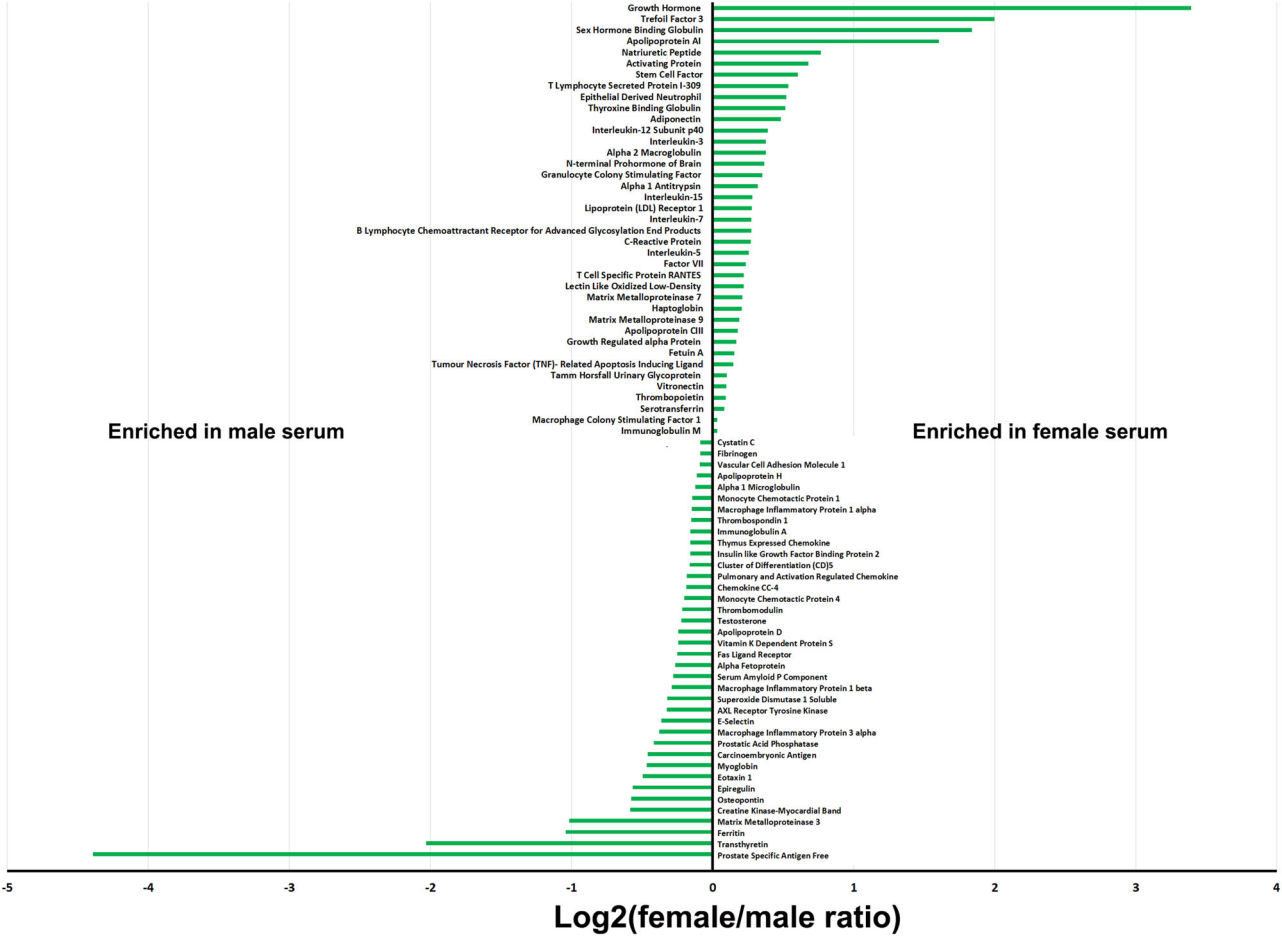
Molecular sex differences in human serum. Log2-scale of female to male serum concentration ratio of 77 analytes (out of 174 identified serum molecules across nine independent cohorts of individuals consisting 196 males and 196 females) that had significant differences in male and female serum concentrations (data for the graph was extracted from reference^44^).

Based on both proteomics and metabolomics data, the composition of female and male plasma differs significantly, with significant variations in the abundance of 231 proteins. Another study analyzed 174 serum biomolecules in 196 male and 196 female human samples, from nine independent cohorts, and probed sex-specific differences^44^. The outcomes of this meta-analysis have revealed robust and reproducible sex differences in 77 of the biomolecules. Among these, 40 have higher concentrations in females (Fig. 2). Sex-specific variations in biomolecules (e.g., hormones) strongly depend on individual physiological condition (Table 1). Several studies have qualitatively probed the role of physiological conditions in plasma protein variations and revealed individual differences in plasma proteome patterns^45–49^. For example, serum concentrations of alpha 1-antitrypsin, alpha 2-HS-glycoprotein, beta 2-glycoprotein III, Gc-globulin, alpha 1-lipoprotein, and alpha 2-AP-glycoprotein decrease in females after menopause, while the concentrations of alpha 1-acid glycoprotein, haptoglobin, serum amyloid P-component, Zn-alpha 2-glycoprotein, beta-lipoprotein, and C1-components increase^45^. As another example, levels of some plasma proteins may change during pregnancy: plasglypican-3 (increases from 10 to 40 weeks), sialic acid-binding immunoglobulin-type lectin-6 (decreases from 10 to 22 weeks and increases from 23 to 40 weeks), placental growth factor (increases from 10 to 31 weeks), prolactin (increases from 10 to 35 weeks), and interleukin-1 receptor 4 (increases from 25 to 40 weeks)^47^.

**Table 1:** Summary of normal hormonal changes in males and females’ serum under different physiological conditions (data extracted from references^126–130^).

Hormone	Female	Male
Hydroxyprogesterone	Follicular <80 ng/dL Luteal <285 ng/dL Postmenopausal <51 ng/dL	Adult <220 ng/dL
Androstenedione	30–200 ng/dL	40–150 ng/dL
Beta-human chorionic gonadotropin (beta-hCG)	Premenopausal nonpregnant: <1.0 U/L Postmenopausal: <7.0 U/L	<1.4 U/L
Calcitonin	≤5 pg/mL	≤10 pg/mL
Dehydroepiandrosterone sulfate (DHEA-S)	44–332 μg/dL	89–457 μg/dL
Estradiol	Follicular: 10–180 pg/mL Mid-cycle peak: 100–300 pg/mL Luteal: 40–200 pg/mL Postmenopausal <10 pg/mL	20–50 pg/mL
Follicle-stimulating hormone	Follicular/luteal: 2–9 mIU/mL (2–9 U/L) Mid-cycle peak: 4–22 mIU/mL (4–22 U/L) Postmenopausal: >30 mIU/mL (>30 U/L)	Adult: 1–7 mIU/mL (1–7 U/L) Children, Tanner stages: 1, 2 0.5–8.0 mIU/mL (0.5–8.0 U/L) Children, Tanner stages: 3, 4, 5 1–12 mIU/mL (1–12 U/L)
β-Human chorionic gonadotropin (β-hCG)	Premenopausal nonpregnant: <1.0 U/L Postmenopausal: <7.0 U/L	<1.4 U/L
Luteinizing hormone (LH)	Follicular/luteal: 1–12 mIU/mL (1–12 U/L) Mid-cycle peak: 9–80 mIU/mL (9–80 U/L) Postmenopausal: >30 mIU/mL (>30 U/L)	Adult: 2–9 mIU/mL (2–9 U/L) Children, Tanner stages: 1, 2, 3 <9.0 mIU/mL (<9.0 U/L) Children, Tanner stages: 4, 5 1–15 mIU/mL (1–15 U/L)
Osteocalcin	7.2–27.9 ng/mL	11.3–35.4 ng/mL
Progesterone	Follicular: 0.02–0.9 ng/mL Luteal: 2–30 ng/mL	Adult: 0.12–0.3 ng/mL
Testosterone	18–54 ng/dL	291–1100 ng/dL

It has been increasingly recognized that plasma composition (e.g., due to individual variations and health status)^42,43,50^ significantly affects NP’s biomolecular/protein corona profiles. Therefore, one can expect that sex-based biomolecular differences in any given plasma sample to be reflected in NP biomolecular/protein corona composition. Although corona profiles have been widely investigated under a variety of conditions^6^, to the best of our knowledge, few reports have considered the effect of sex on the composition and decoration of biomolecular/protein corona. For example, it has been demonstrated that incubation of identical 70-nm SiO_2_ NPs with plasmas of male and female zebrafish leads to the formation of coronas with different compositions;^51^ the vitellogenin family (egg yolk precursor proteins with high plasma concentrations in female fish^52,53^) was found to represent one major difference in male and female corona compositions^51^. Blood cells therefore interact differently with NPs exposed to male and female plasma. The NPs with coronas containing proteins present in female plasma are preferentially recognized by leukocytes compared to their male counterparts. This finding suggests that blood circulation time and pharmacokinetics of identical NPs, together with the host immune system response to them, may differ significantly between males and females.

### Sex-dependent toxicity and therapeutic efficacy of NPs

#### Sex-specific molecular and cellular structures

One of the main challenges to the vigorous study of NP interactions with biosystems is the selection of appropriate cells that originate at a very early stage of development, prior to the initiation of hormonal changes and genetically and structurally driven sexual dimorphisms. The use of embryonic cells is one strategy to address this issue. For example, male and female mouse embryonic stem cells (mESCs) have been used to determine the sex-specific effects of silver NPs on stem cell differentiation^54^. Low concentrations (0.2, 0.5, and 1.0 μg mL^−1^) of silver NPs delay the differentiation of female mESCs by interfering only with the X chromosome inactivation process. The same NPs, however, show no interference with the self-renewal process and exert no significant cytotoxic effect. It is well understood that even slight interference in X chromosome inactivation considerably impairs programmed ESC differentiation by regulating the expression of relevant genes^55^. The random inactivation of one X chromosome of the female cell is known to equilibrate gene content between the sexes^56,57^. Xist and Tsix are noncoding RNAs playing critical roles in silencing and activating the X chromosome, respectively. A low concentration of silver NPs interferes with the expression of Xist and Tsix and disrupts the optimal Xist/Tsix ratio for X chromosome inactivation. Silver NPs also prevent the expression of genes responsible for triggering programmed ESC differentiation by enhancing the trimethylation of histone (histone 3 lysine 27 trimethylation, H3K27me3). Therefore, even a low concentration of silver NPs can cause a series of transcriptome and epigenome changes interrupting X chromosome inactivation and subsequent ESC differentiation in females. The most striking observation has been that such epigenome and transcriptome changes are absent in male mESCs treated with low concentrations of silver NPs, revealing programmed differentiation even after long exposure to silver NPs. As X chromosome inactivation is a female cell-specific phenomenon, agents interfering with this process impair the differentiation of female cells only^54^.

As another example, human amniotic stem cells (hAMSCs), one of the earliest sources of somatic stem cells, have been employed to probe the effect of cell sex on NP uptake^17^. Exposure to quantum dots (QDs) reflects significant sex-dependent differences in cellular uptake efficacy in human amniotic stem cells (hAMSCs): female cells take up more QDs than male cells. Aside from sex-dependent biomolecular/protein coronas, sex-based differences in the structure and function of cells also play key roles in their response to NPs. It was shown that sex-dependent uptake of QDs is caused by differences in arrangement, shape, and distribution of actin filaments (cytoskeleton), which regulate the endocytosis and cellular trafficking of QDs. Remarkably, male and female cells show different capabilities in reprogramming hAMSCs into pluripotent stem cells when treated with nanosized Sendai virus commonly used for the preparation of pluripotent stem cells. The Sendai virus shows more effective transfection—and hence, better reprogramming efficacy—into female hAMSCs compared to male hAMSCs^17^.

#### Sex-specific biomolecules

For accurate evaluation of the interactions of NPs with non-embryonic cells, the possible roles of sex-specific hormonal effects on both cell behavior/characteristics and biomolecular/protein corona of NPs should be considered in detail. Hormones are sex-specific chemical messengers regulating the duration and level of crucial processes (e.g., growth, development, metabolism, and reproduction) in tissues and organs^58,59^. Therefore, small variations in hormone production/balance may produce significant changes in susceptibility/resistance to diseases and therapeutic drugs/NPs. For example, it is well understood that cell sex characteristics and sex hormones have significant impacts on both normal lung physiology and lung diseases^60^. New findings from the NELSON Trial provide evidence of sex-specific differences in lung cancer screening and survival^61^. Notably, the results of related research suggest that lung cancer progression may be more rapid and lethal in females. These important factors need to be considered in interpretation of the toxicity and therapeutic outcomes of NPs being used for tissues (e.g., lung) that are highly affected by sex-specific phenomena. In addition, sex-specific disease-dependent biomolecular changes in plasma can significantly affect the biomolecular/protein corona profiles of NPs and thus their interactions with biosystems. For example, different concentrations of vitellogenin in male and female zebrafish plasma significantly affected the composition of the corona formed on the surface of SiO_2_ NPs^51^. In addition, since the protein metabolome profiles of males and females reflect considerable differences in amino acids, fatty acids, and lipids^62^, their blood plasmas also have different metabolome and lipidome profiles. Depending on the sex and age of a person, different metabolic pathways are activated and different plasma metabolite association networks form^63^. As the metabolome can affect protein-NP interactions^64^, one could expect metabolomics variations to affect the biomolecular/protein corona composition of NPs in a sex-specific manner.

#### Sex-specific immunity

Immune system response to NPs is an important factor in their toxicity and therapeutic efficacy; and sex-related physiologic differences significantly affect host immune system responses to foreign materials including NP/biomolecular therapeutics. In rodents, for example, the phagocytic activity of macrophages is stronger in females than in males^65^. Indeed, female mice and rats have more CD45^+^ leukocytes and macrophages in both naive peritoneal and pleural cavities^66^. In the respiratory tract, sex hormones including estrogens, androgens, and progesterone regulate the number and function of innate immune cells in a sex-specific manner^67^. Sex can also affect patient responses to cancer immunotherapy^23^. Sex-specific differences have also been observed in the functions of other immune cells such as T-lymphocytes, B-lymphocytes, astrocytes (in the brain), and natural killer cells^20,68^. Therefore, males and females show different innate/adaptive immune responses to antigens/infections. The immune response to self-antigens, leading to autoimmune diseases, is more prevalent in females; ~80% of patients diagnosed with autoimmune disease are females^69^. As stated above, it is legitimate to hypothesize that immune system responses to identical NPs are sex-dependent. For example, female mice have stronger immune responses (e.g., through significant pathological changes in spleen and thymus index) to PEG-coated gold NPs compared to male mice^12^. In addition, male mice treated with gold NPs show more significant infection and inflammation compared to female mice^12^.

The immune system plays a critical role in many diseases, including cancers, in a sex-specific manner. Due to their genetic and epigenetic mosaicism due to random inactivation of one of the X chromosomes in XX female cells, females are more resistant and show stronger immune response to cancer compared to males^70^. In addition, in some cancers such as glioblastoma, current treatments show better outcomes in females than in male patients^71^. For example, the effectiveness of immune checkpoint inhibitors (e.g., antibodies against CTLA-4, PD-1, and PDLA-1), among the most efficient therapeutics, is sex-dependent^72–74^. One of the recent promising applications of NPs in cancer is immunotherapy^75^; here again, sex may have significant effects on the therapeutic efficacy of nanomedicine products, in an organ-dependent manner, for several reasons, including sex-related differences in tumor microenvironments^76^; sex-associated molecular differences in response to cancer immunotherapy;^77^ and the role of NPs in the efficacy of the immune system in tumor microenvironments^78^. For example, it was shown that ferumoxytol, a Food and Drug Administration (FDA)-approved iron supplement, could increase the number of pro-inflammatory M1 tumor-associated macrophages in female mice, demonstrating intrinsic inhibitory effects on the growth of early mammary cancers together with lung cancer metastases in liver and lungs^78^. Although the study was limited to female mice, one may expect to observe different therapeutic outcomes with ferumoxytol in male mice, as the production of cytokines and chemokines by macrophages differs between males and females^79^.

#### Sex-specific disease environments

NPs not only exert different toxic effects in males and females, but also show sex-dependent variations in therapeutic efficacy. The fetal hypoxia that often occurs during pregnancy may induce oxidative/nitrosative stress, which increases the risk of catastrophic diseases in mature offspring^80^. The placentas of male and female fetuses use different mechanisms to suppress oxidative stress and show different responses to identical stimuli. NPs encapsulating MtiQ, an antioxidant suppressing mitochondrial oxidative stress, have been used to treat fetal hypoxia and prevent subsequent placental dysfunction in animal models, showing sex-specific therapeutic effects^81^ in favor of female fetuses. Therefore, sex differences should be considered throughout the development of nano-based drugs for treatment of placenta- or embryo-related disorders.

Since sex differences affect the prevalence and progression of many diseases^82–84^, differences in therapeutic efficacies of drug/NPs between males and females should be considered in an organ-specific manner. This is because male and female cells have different sex chromosomes, display dissimilar gene expression patterns, and activate distinct sex-specific signaling pathways in response to the same treatment^11,85,86^. For example, the body’s natural healing process in response to cardiac diseases (e.g., cardiomyopathy) is sex specific^87–89^. Although nanotechnologies have been widely used to address cardiovascular diseases^90,91^, the role of sex in therapeutic efficacy has not been critically considered. It is noteworthy that even circulating biomarkers and/or biomolecules associated with cardiovascular diseases are sex dependent^92^, which may affect the biomolecular/protein corona of therapeutic NPs. Such variation in circulating biomolecules may also be related to the gut microbiota^93^, whose composition depends strongly on host sex^94^. Although NP-based oral treatments may affect the composition of the gut microbiome in a sex-dependent manner (e.g., the significant effects of silver NPs on the gut microbiome of male but not female zebrafish)^19^, we do not consider them in this review.

Some injuries may also have sex-specific physiological effects that may significantly affect NP delivery to the injured tissue. For example, it was shown that within the first couple of hours following traumatic brain injury (TBI), the blood–brain barrier (BBB) often undergoes less damage in females than in males^95,96^. This will significantly affect the delivery of identical NPs to the brain tissue in a sex-dependent manner. It has been demonstrated in transgenic mice models of TBI that PEGylated polystyrene NPs were more effectively delivered to female than to male brain tissue^96,97^. Although not evaluated in previous studies, the therapeutic efficacy of the NPs that accumulate in brain tissue is assumed to be sex-specific, at least in part due to the sex-specific response by brain immune cells. Recent reports have revealed significant differences in function, morphology, abundance, and gene expression profiles between male and female microglia, which are the main immune cells involved in central nervous system maturation, signal transduction, and clearance of protein/cell aggregates/debris in the brain^98–101^. As sex differences in microglia are involved in the cause of differing incidence and prevalence of neurodegenerative diseases such as Alzheimer’s, Parkinson’s disease, Amyotrophic lateral sclerosis, and frontotemporal dementia between male and female patients, the successful use of therapeutic nanomedicines for these diseases must also be sex-specific^102–105^. Microglia are the resident immune cells in the brain and maintain their homeostasis and functionality. The role of sex in microglial cell functions and its effect on physiological and pathological conditions in the brain has been discussed in a recent review article by Yanguas-Casás^106^.

### Challenges and recommendations in considering sex in nanomedicine studies

**Fig. 3:**
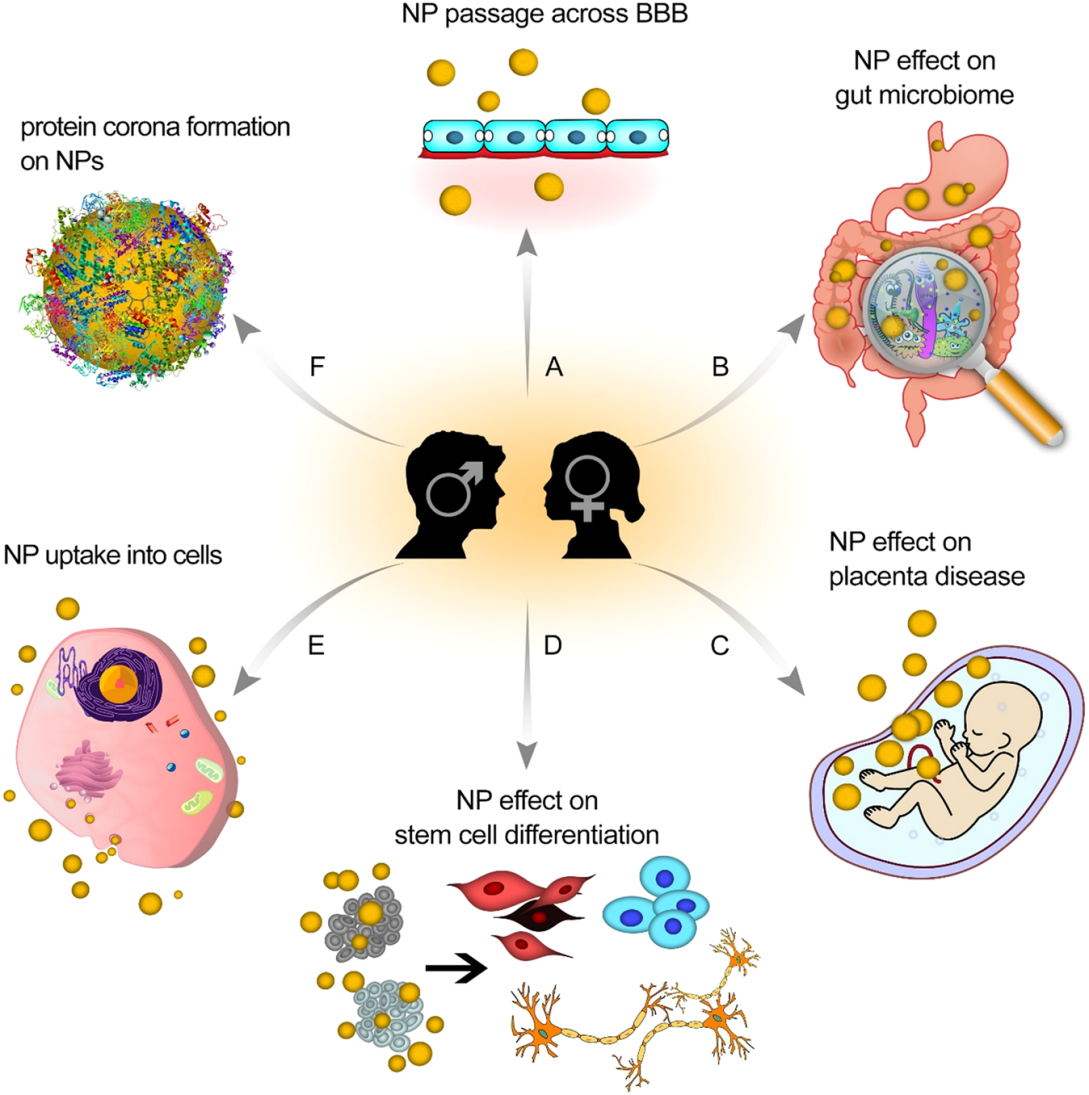
Schematic representation of sex-dependent therapeutic/toxic effects of nanoparticles. **A** Crossing the blood–brain barrier, (**B**) gut microbiome composition, (**C**) placenta-related diseases, (**D**) stem cell differentiation, (**E**) uptake of NPs into cells, (**F**) formation of biomolecular/protein corona.

Taking sex into account will have a significant impact on the success of both laboratory and clinical research in nanomedicine, encompassing increased safety and efficacy, identifying sex differences in cellular characteristics, plasma composition, and secreted factors that affect NPs’ biomolecular/protein coronas and functions (e.g., in terms of their safety and therapeutic efficacies; Fig. 3). It is clear that many researchers in the field of (nano)medicine are aware of the importance of considering sex in their studies; there are, however, many challenges to overcome before the importance of sex can be systematically considered in studies and/or reports. In this section, we outline the central challenges and propose possible strategies to address them.

### Physiological complexity of considering sex

#### Description of the challenge

Compared to male biosystems, female biosystems (e.g., plasma composition) have more physiological complexity, due to conditions such as menstruation, pregnancy, lactation stage, and menopause, among others. These complexities may make the outcomes of studies involving females less reliable and/or reproducible than the findings of studies on males. Age also needs to be properly considered in study designs, as many sex-specific hormones are strongly dependent on age and individual health status. For example, during puberty, hormones differently induce the development of sex organs in males and females^107^. Menopause is characterized by reduced concentrations of progesterone and estrogens and increased levels of follicle stimulating hormone (FSH)^108^; premenopausal and postmenopausal females have different hormone profiles^108^; and blood concentrations of progesterone, estriol, and prolactin change considerably during pregnancy^108^. All these variables may have significant effects on the biomolecular/protein corona profile of NPs and their interactions with biological systems.

#### Mitigation strategies

To obtain useful biomolecular/protein corona data, researchers need to have full information on the age, sex, health, and physiological condition of plasma donors. Researchers should avoid using pooled plasma and/or plasma with no comprehensive information on the donor (except the personal information), as the outcomes may not be conclusive. When seeking to determine the role of sex in the biomolecular/protein corona profile of NPs, the characteristics/specification of plasma donors (e.g., age, health, and physiological conditions, and ethnicity) need to be the same for both sexes. In addition, other influential factors (e.g., blood collection and reservation tubes, duration, and temperature of plasma storage) need to be carefully considered in the experimental setup. The best approach to considering the effect of female-specific physiological conditions (e.g., menstruation, pregnancy, lactation stage, and menopause) on the biological efficacy of NPs is to use plasma samples from the same individuals in a prospective manner.

### Biosystems

#### Description of the challenge

Results published from both in vitro and in vivo studies should include full details on the sex of the cells and/or animals. Unfortunately, in most studies, the sex of cell lines used in in vivo and in vitro (e.g., for tumor implantation) are not appropriately considered and reported in the current literature^6^. Even in the limited number of publications that do report cell sex, other crucial information (e.g., number of the passage)^36^ are often missing. The number of cell passages is critically important, as some male cell lines widely used in in vitro studies gradually lose their Y chromosome with increasing numbers of passages^109^.

#### Mitigation strategies

Researchers need to have full information about the cells they intend to use for in vitro and/or in vivo studies, including their sex, origin, passage numbers, and storage conditions. In addition, to completely investigate the effect of sex on cellular responses to NPs. The selected male and female cells should (i) have the same origin (e.g., organs and species), (ii) have been extracted from similar donors (e.g., in terms of age, health condition, and ethnicity), and (iii) have had minimal exposure to sex-specific hormones. For in vitro evaluations of the effects of sex on NPs, researchers must ensure that their culture media are free from sex-specific hormones that may differentially affect the behavior of male and female cells. Another major problem is the limited number of comparable male and female cells offered by vendors/distributors of cells [e.g., The Global Bioresource Center (ATCC)]. Therefore, efforts by bioresource companies, as important stakeholders, should be made to provide resources for studying sex as an important biological variation. In the absence of such resources, researchers may have to access suitable cells through their clinical collaborators.

### Purity of biomolecular/protein corona

#### Description of the challenge

As discussed above, achieving rigorous and concise information on the composition of biomolecular/protein corona is essential to predict NPs’ interactions with biosystems and their biological impact. Recent findings obtained by combining cryo-electron microscopy, cryo-electron tomography, and image simulation have revealed that the biomolecular/protein corona formed on the surface of NPs contain some impurities in the form of non-specific clusters of biomolecules. This will influence the accuracy of the results of proteomics analysis of the biomolecular/protein corona^110^. Although the mechanism formation of these clusters and their function is unknown, the composition and concentration of these impurities associated with the corona formed by exposure of NPs to male and female plasma could be different.

#### Mitigation strategies

More in-depth attention needs to be paid for investigation of the purity of protein corona prior proteomics analysis. Another crucial aspect is experimental obstacles affecting the characterization of NPs and their corona profiles. Using diluted NPs to study biomolecular/protein corona is one practical approach that can minimize such impurity issues^110,111^.

### Access to biological resources

#### Description of the challenge

Access to materials and data are easily available through extensive studies of differences between the sexes on the reproductive systems. Lack of availability of resources on sex differences in nonreproductive biology, where differences are much less recognized, creates critical challenges in the field. Although most of the differences in sex in nonreproductive areas of biology are difficult to detect, some of these differences can have significant implications for clinical and laboratory research within the discipline of nanomedicine. An additional problem is the fact that many researchers may not have access to comparable male and female biosystems (e.g., cells, plasmas, animals) for a variety of reasons, including lack of a clinical collaborator/center.

#### Mitigation strategies

Researchers should thoroughly report all available information, including sex, on the biosystems they study and be specific about their claims/conclusions. For example, if studies are performed on male plasma and/or cells, that should be clearly conveyed in an article’s conclusion to avoid apparently conflicting results arising from sex-specificity; that is, the results might not be reproducible if female plasma and/or cells are used by other researchers.

### Conclusions and future perspectives

Beyond conventional medicine and commercially available parent drugs, therapeutic and diagnostic applications of nanomedicine have yet to be developed to the point of clinical use against devastating diseases such as cancer, heart disease, and neurological diseases. Among the reasons for slow progress in many areas of therapeutic nanomedicine are the lack of comprehensive, basic scientific research and an insufficient understanding of the mechanisms involved in cellular interactions with nanomaterials and functionalized NPs together with other complex biological variables, including sex, age, race, health status, and comorbidities^6^.

To accelerate successful clinical translation of diagnostic and therapeutic nanomedicine, future studies should consider the importance of overlooked factors described in this review and report critical information including the alteration of physicochemical and biological properties of NPs upon exposure to body fluid and most importantly, the significance of biological variables including sex. More specifically, to robustly consider and understand the role sex plays in the safety and therapeutic efficacy of nanomedicines. There is a need for advancement of our understanding of (i) the effects of ethnicity, disease stage, age, sex, and physiological conditions on the biomolecular/protein corona profiles of NPs, (ii) the interaction of NPs with sex-specific elements of the immune system (e.g., macrophages, T-cells, B-cells and natural killer cells), and (iii) the interactions of NPs with sex-specific disease environments (e.g., for cancer: tumor microenvironment, tissue, cellular components, and biochemical environment). As different laboratories may have significant variations in the characteristics readouts of identical NPs^112^, interlaboratory procedures and instrumentations should be standardized for assessment of the role of sex on diagnostic and therapeutic nanomedicine.

The importance of the topics outlined in this article is evident by the application of nanomedicine-based approach to develop and produce an effective vaccine against COVID-19. According to the initial reports^113,114^, the NP-based vaccines developed by Pfizer/BioNTech and Moderna performed slightly better in males [95.4% (Moderna) and 96.4% (Pfizer/BioNTech)] than in females [93.1% (Moderna) and 93.7% (Pfizer/BioNTech)]^113,114^. While monitoring of the sex-specific efficacy outcomes of these vaccines should be continuously monitored and reported, more fundamental studies are required to be conducted on the sex-specific efficacy of the NP-based vaccines at cellular and molecular level. The outcomes of such efforts will help the scientific community to better define potential sex-dependent therapeutic/toxic effect of COVID-19 NP-based vaccines which in turn pave a way for development of sex-specific vaccine design, development, and administration.

The other important factor that should be considered in the sex-specific meta-analysis of nanomedicine is to minimize publication bias (i.e., “the null results of studies may face a higher barrier to publication than those that yield statistically significant differences.”^115^)^1,19^. When only statistically significant results are published, it is obvious that the outcomes misrepresent the real findings^116^. According to Joober and co-workers^117^, “withholding negative results from publication—publication bias—could have major consequences for the health of millions”. The negative impact of publication bias has been studied and validated in most of the scientific fields including chemistry, medicine, and biomedical sciences^118–123^. Due to the multidisciplinary nature of nanomedicine, it is obvious that nanomedicine literature has the same issue which has been poorly considered so far^124^; despite extensive number of publication since its emergence, addressing the literature bias in nanomedicine has been ignord^124^.

Overall, this review suggests that the sex of biosystems (e.g., cells and plasmas) should be considered, as an essential parameter, in nanomedicine studies. Such consideration will be critical in addressing the reasons behind the alarming signals of failure in therapeutic cancer nanomedicine^7^.

A collective national and international effort among all involved stakeholders (e.g., basic scientists, clinician, pharmaceutical, and grant agencies) is required to introduce policies, guidelines and regulation to promote inclusion of sex-specific factors in nanomedicine research. A good example is the initiation of the Committee on Understanding the Biology of Sex and Gender Differences that was constructed by the National Academy of Science to address biology at the cellular, developmental, organ, organismal, and behavioral levels with emphasis on Sex and Gender Differences. The results of this comprehensive study have been published in a book by T.M. Wizemann and M-L. Pardue^125^. Unfortunately, most of the recommendations outlined in this book have been ignored so far. Such a framework of integrated responding of all involved stakeholders may increase responsibility and response-ability in understanding and solving problems in timely and efficient manner^126^. Considering the current challenges facing nanomedicine, it would be helpful to establish a similar undertaking to overcome some of the shortfalls in nanomedicine. Inclusion of sex-specific biological factors at cellular and molecular is essential for advancements of nanobiotechnology and nanomedicine for both sexes. Undoubtedly, nanomedicine holds the promise to improve the health care system in near future; therefore, it is important for the scientific community to pay more attention to the importance of sex differences in their studies. Last, but not least, if we are to move closer to the lofty goal of personalized nanomedicine, the role of gender must also become a central consideration in future studies.
